# Computed Tomography-Based Morphometric Analysis of Ossification Centers of Lesser Wings of Sphenoid Bone in Human Fetuses

**DOI:** 10.3390/brainsci15060558

**Published:** 2025-05-23

**Authors:** Magdalena Grzonkowska, Michał Kułakowski, Mariusz Baumgart

**Affiliations:** 1 Department of Normal Anatomy, The Ludwik Rydygier Collegium Medicum in Bydgoszcz, The Nicolaus Copernicus University in Toruń, 87-100 Toruń, Poland; mariusz.baumgart@cm.umk.pl; 2 Powiślański University in Kwidzyn, 82-500 Kwidzyn, Poland; 3 Clinical Department of Orthopedics and Traumatology, Jan Biziel University Hospital nr 2 in Bydgoszcz, The Nicolaus Copernicus University in Toruń, 87-100 Toruń, Poland; mkulakowski@poczta.fm

**Keywords:** fetus, ossification center, sphenoid bone

## Abstract

**Objectives**: The aim of the present study was to examine the growth dynamics of the ossification centers of the lesser wings of the sphenoid bone in the human fetus based on linear, planar, and volumetric parameters. **Methods**: The examinations were carried out on 37 human fetuses of both sexes (16 males and 21 females) after 18–30 weeks of gestation. These were obtained from spontaneous miscarriages and preterm deliveries. Using computed tomography (CT), digital image analysis software, 3D reconstruction, and statistical methods, the size and growth patterns of the ossification centers of the lesser wings of the sphenoid bone were evaluated. **Results**: All morphometric parameters—length, width, projected surface area, and volume—of the ossification centers of the lesser wings of the sphenoid bone increased proportionally with gestational age. No significant sex-related or lateral differences were observed. **Conclusions**: The numerical data obtained from CT-based analysis and the observed growth trends of the ossification centers of the lesser wings of the sphenoid bone may serve as age-specific normative references. These findings may support clinicians—including anatomists, radiologists, obstetricians, pediatricians, and craniofacial surgeons—in the assessment of normal fetal cranial development and the early diagnosis of congenital craniofacial anomalies.

## 1. Introduction

The sphenoid bone plays a pivotal role as an anatomical landmark within the cranial structure, owing to both its complex morphology and its proximity to several critical anatomical components of the head. This bone shields several vital brain structures, such as the pituitary gland, the optic chiasm, and the internal carotid artery. Moreover, the sphenoid bone forms conduits for the passage of five cranial nerves: the optic, oculomotor, trochlear, trigeminal, and abducens nerves. As a result, any pathology, injury, or disorder affecting the sphenoid bone can lead to a broad spectrum of neurological symptoms and functional impairments [1].

Within the sphenoid bone’s body lie the sphenoidal sinuses, which are two air-filled cavities arranged symmetrically. Infections or inflammatory conditions affecting these sinuses may manifest as headaches, facial pain and, in some cases, complications involving adjacent anatomical structures. In neurosurgical practice, the sphenoid bone serves as a critical access point, particularly during transsphenoidal procedures, which are widely utilized surgical approaches for the treatment of pituitary tumors [1].

The process of the ossification of the human cartilaginous skull begins during the embryonic period and involves approximately 110 ossification centers. These centers contribute to the formation of 45 cranial bones in the neonate, which gradually fuse after birth, ultimately reducing the number of cranial bones to 22 in adults. Despite the completion of the primary ossification process, remnants of cartilaginous tissue can still be observed in certain regions of the skull, indicating prolonged cranial development that continues until approximately 20 years of age [2,3].

Although the ossification process of individual cranial bones has been extensively described, the morphometric assessment of the ossification centers of the lesser wings of the sphenoid bone in human fetuses has not yet been performed thoroughly. The aim of the present study is to conduct—for the first time in the literature—a morphometric analysis of the ossification centers of the lesser wings of the sphenoid bone in human fetal specimens, utilizing computed tomography-based imaging.

This study aimed to achieve the following objectives:

- Investigate potential sex-related and lateral differences in all examined parameters;
- Perform morphometric analysis of the ossification centers of the lesser wing of the sphenoid bone in human fetuses (including linear, planar, and volumetric parameters) in order to establish normative value ranges;
- Assess the growth dynamics of all analyzed parameters and to develop mathematical models with the best fit.

## 2. Materials and Methods

### 2.1. Examined Sample

The present study was based on a sample of 37 human fetal specimens (16 male and 21 female), with gestational ages ranging from 18 to 30 weeks, obtained following spontaneous miscarriages and preterm deliveries. All specimens, collected prior to the year 2000, were part of the anatomical collection curated by the Department of Normal Anatomy at the Collegium Medicum in Bydgoszcz, Nicolaus Copernicus University in Toruń. Ethical clearance for the study was obtained from the Bioethics Committee of the Ludwik Rydygier Collegium Medicum in Bydgoszcz (KB 275/2011). All research activities were performed in compliance with applicable Polish legislation, within the framework of the institutional Body Donation Program for adults and fetuses, and conformed to the ethical principles articulated in the Declaration of Helsinki.

The morphometric analysis was carried out over the course of October 2024 at the Department of Anatomy, Ludwik Rydygier Collegium Medicum, Nicolaus Copernicus University in Toruń. Only fetal specimens exhibiting well-preserved anatomical features and accompanied by complete clinical documentation were included in the study. Cases demonstrating overt morphological anomalies or musculoskeletal developmental disorders were excluded. Accordingly, specimens affected by congenital malformations, intrauterine growth restriction, or other significant developmental abnormalities were not considered for analysis.

Fetal age was assessed using crown–rump length (CRL) measurements in conjunction with the gestational age, calculated from the date of the mother’s last menstrual period. Inclusion in the study was limited to specimens demonstrating a high degree of concordance between these two methods of age estimation (R = 0.98, *p* < 0.001). A comprehensive overview of the study population, including gestational age, sample size, and sex distribution, is provided in Table 1.

### 2.2. Morphometric Measurements and Assessment of Ossification Centers

**Figure 1:**
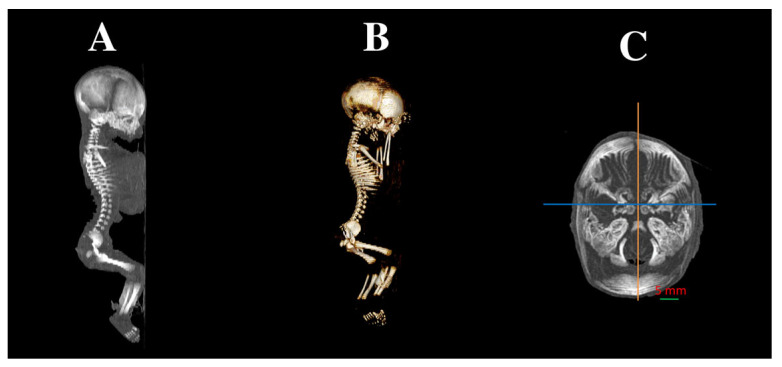
Computed tomography image of whole female human fetus at 25 weeks of gestation presented in sagittal view (**A**), accompanied by three-dimensional reconstruction in same projection (**B**), and cranial CT image in transverse plane (**C**).

**Figure 2:**
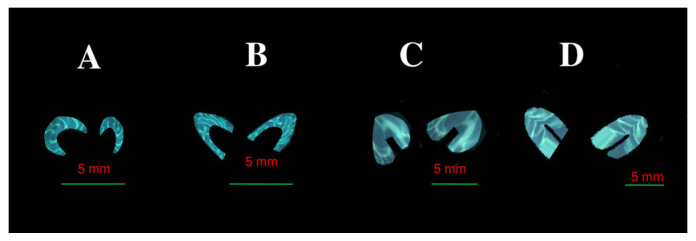
Visualization of volume of ossification centers of lesser wings of sphenoid bone (month V—(**A**), VI—(**B**), VII—(**C**), VIII—(**D**)).

High-resolution fetal scans were acquired in DICOM format at 0.4 mm intervals using a Siemens Biograph 128 mCT scanner (Siemens Healthcare GmbH, Erlangen, Germany), located in the Department of Positron Emission Tomography and Molecular Imaging, Oncology Center, Collegium Medicum, Nicolaus Copernicus University in Bydgoszcz, Poland (Figure 1 and Figure 2).

The gray scale values of the acquired CT images, expressed in Hounsfield units (HU), ranged from −275 to −134 for the minimum, and from +1165 to +1558 for the maximum. Consequently, the window width (WW) varied between 1404 and 1692, while the window level (WL) ranged from +463 to +712. Imaging parameters were as follows: tube current (mAs)—60, tube voltage (kV)—80, pitch—0.35, field of view (FoV)—180, and rotation time—0.5 s. The CT data were reconstructed using a slice thickness of 0.4 mm, an image increment of 0.6 mm, and a B45 f-medium kernel.

**Figure 3:**
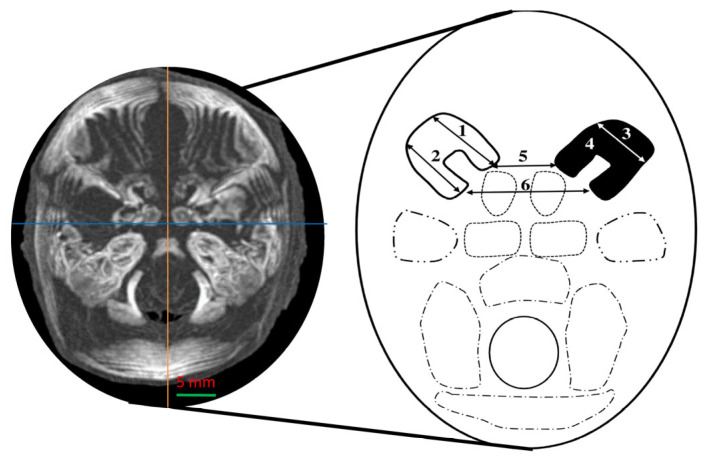
Diagram illustrating measurement protocol for ossification centers of lesser wings of sphenoid bone.

Measurements of the ossification centers of the lesser wings of the sphenoid bone were conducted in accordance with a standardized imaging protocol (Figure 3). The sagittal, vertical, and transverse axes were oriented orthogonally to one another, allowing for the precise measurement of the length and width of both ossification centers within the transverse plane—defined as the plane exhibiting the greatest projection surface area.

For each fetus, the analysis of the linear dimensions, projected surface areas, and volumes of the ossification centers of the lesser wing of the sphenoid bone was conducted. Despite the presence of a cartilaginous stage, the morphometric assessment of sagittal and transverse dimensions, as well as volume, was feasible, as the contours of the entire bone were already clearly distinguishable [4,5].

Measurements of the ossification centers of the lesser wings of the sphenoid bone included the following parameters:

1–2. The length of the anterior and posterior portions—this was determined by measuring the distance between the initial and terminal margins of the ossification center in the transverse plane (Figure 3).

3. Width—this measured as the distance between the initial and terminal lateral margins of the ossification center in the transverse plane (Figure 3).

4. Projected surface area (right and left)—this was determined by outlining the ossification center of the lesser wing of the sphenoid bone in the transverse plane (Figure 3).

5–6. Distance between the anterior and posterior parts—this was measured between the proximal margins of the anterior and posterior segments on both the right and left sides (Figure 3).

7. Volume—this was calculated using advanced imaging analysis tools, enabling three-dimensional reconstructions that accounted for the location and radiodensity of the ossified tissue (Figure 2).

### 2.3. Statistical Analysis

In the current study, statistical analyses were performed using Statistica 12.5 and PQStat 1.6.2 software packages. All numerical data underwent comprehensive statistical evaluation. The distribution of variables was verified using the Shapiro–Wilk test (W), while the homogeneity of variances was assessed with Fisher’s F-test. For the comparison of means, Student’s *t*-test was applied to both dependent variables (left vs. right) and independent variables (male vs. female). When comparing more than two groups, one-way analysis of variance (ANOVA), followed by Tukey’s post hoc test, was employed. In instances where the assumption of homogeneity of variance was violated, the non-parametric Kruskal–Wallis test was utilized.

The developmental dynamics of the analyzed parameters were evaluated using both linear and nonlinear regression models. The goodness of fit of the estimated curves relative to the observed data was determined based on the coefficient of determination (R^2^). Statistical significance was set at a threshold of *p* < 0.05. Associations between variables were examined using Pearson’s correlation coefficient (r).

Each measurement was performed three times under standardized conditions, and the mean value of the recorded results was calculated. As presented in Table 2, the intra-class correlation coefficients (ICCs), determined from repeated measurements conducted by a single observer (M.G.), were statistically significant (*p* < 0.001) and indicated excellent measurement repeatability.

## 3. Results

The mean values and standard deviations of the measured parameters of the ossification centers in the right and left lesser wings of the sphenoid bone in human fetuses, corresponding to the analyzed stages of development, are summarized in Table 3, Table 4 and Table 5.

Statistical analysis found no significant differences related to sex or laterality, thereby justifying the use of a unified growth curve for each of the assessed parameters.

The developmental progression of the dimensions of the ossification centers in the lesser wings of the sphenoid bone exhibited a linear pattern, reflecting a direct proportional relationship with fetal age.

### Morphometric Parameters of the Ossification Centers of the Lesser Wings of the Sphenoid Bone

**Figure 4:**
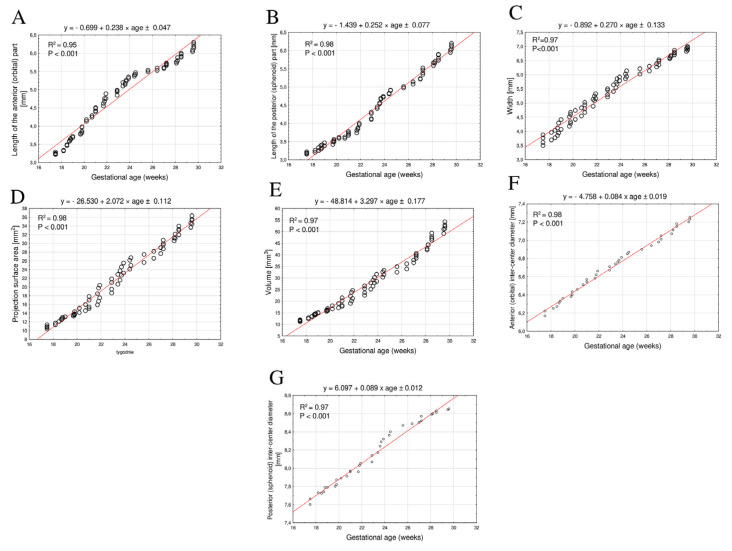
Regression lines for length (**A**,**B**), width (**C**), projected surface area (**D**), volume (**E**), and anterior and posterior inter-center diameters (**F**,**G**) of ossification centers of lesser wings of sphenoid bone.

The mean length of the anterior part of the ossification center of the lesser wing of the sphenoid bone between 18 and 30 weeks of gestation ranged from 3.19 ± 0.03 mm to 6.05 ± 0.10 mm on the right side, and from 3.22 ± 0.04 mm to 6.11 ± 0.10 mm on the left side. The growth pattern followed a linear model, where y = −0.699 + 0.238 × age ± 0.47, with R^2^ = 0.95 (Figure 4A).

The mean length of the posterior part of the ossification center of the lesser wing of the sphenoid bone within the same gestational age range varied from 3.29 ± 0.05 mm to 6.19 ± 0.11 mm on the right side, and from 3.26 ± 0.05 mm to 6.15 ± 0.11 mm on the left side, following a linear function, where y = −1.439 + 0.252 × age ± 0.077, with R^2^ = 0.98 (Figure 4B).

The mean width of the ossification center of the lesser wing of the sphenoid bone between 18 and 30 weeks of gestation increased from 3.6 ± 0.01 mm to 6.96 ± 0.04 mm on the right side, and from 3.87 ± 0.10 mm to 6.87 ± 0.05 mm on the left side. The growth followed a linear function, where y = −0.892 + 0.270 × age ± 0.133, with R^2^ = 0.97 (Figure 4C).

The mean projected surface area of the ossification center of the lesser wing of the sphenoid bone increased from 10.81 ± 0.47 mm^2^ to 35.53 ± 0.84 mm^2^ on the right side, and from 11.32 ± 0.39 mm^2^ to 34.48 ± 0.82 mm^2^ on the left side, also demonstrating a linear growth pattern, where y = −26.530 + 2.072 × age ± 0.112, with R^2^ = 0.98 (Figure 4D).

The mean volume of the ossification center of the lesser wing of the sphenoid bone between 18 and 30 weeks of gestation increased from 11.79 ± 0.62 mm^3^ to 52.70 ± 1.61 mm^3^ on the right side, and from 12.12 ± 0.70 mm^3^ to 50.92 ± 1.59 mm^3^ on the left side. The growth followed a linear pattern proportional to fetal age, described by this function, where y = −48.814 + 3.297 × age ± 0.177, with R^2^ = 0.97 (Figure 4E).

The distance between the anterior parts of the ossification centers of the lesser wings of the sphenoid bone increased from 6.21 ± 0.04 mm to 7.23 ± 0.03 mm between 18 and 30 weeks of gestation. The growth followed a linear function, where y = 4.758 + 0.084 × age ± 0.019, with R^2^ = 0.98 (Figure 4F).

The distance between the posterior parts of the ossification centers of the lesser wings of the sphenoid bone increased from 7.66 ± 0.07 mm to 8.65 ± 0.01 mm within the same gestational age range. The growth pattern followed a linear model, where y = 6.097 + 0.089 × age ± 0.012, with R^2^ = 0.97 (Figure 4G).

## 4. Discussion

**Figure 5:**
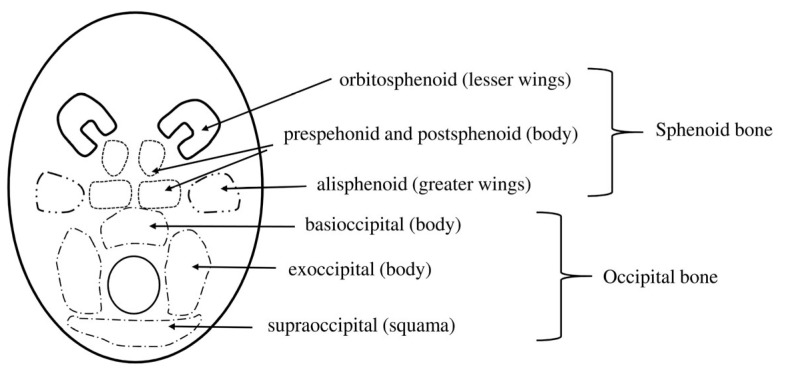
The development of the cranial base.

The full development of the primary cranial base, or chondrocranium—including the sphenoid bone—is achieved by the end of the third month of fetal life. The ossification of the cranial base follows an orderly sequence, beginning in the posterior region and progressing anteriorly. This process occurs in a well-defined order, involving the occipital, sphenoid, temporal, ethmoid, and the orbital parts of the frontal bone [3,6,7,8,9,10], respectively (Figure 5).

However, it is important to note that the onset and rate of ossification do not always correlate precisely with gestational age; in some fetuses, the process may begin earlier and progress more rapidly, while still maintaining the established developmental sequence [3].

The development of the sphenoid bone is of considerable interest from both ontogenetic and evolutionary standpoints. Ontogenetically, the sphenoid originates from two anatomically distinct regions, separated by the sella turcica, with the anterior and posterior components exhibiting differential growth dynamics. From an evolutionary perspective, the posterior portion of the cranial base is regarded as a highly conserved structure, present in early mammalian forms, whereas the anterior portion is considered an evolutionarily derived feature that plays a pivotal role in guiding the characteristic pattern of facial growth in humans [11].

It is essential to underscore that the development of the sphenoid bone represents a highly complex and coordinated process. While ossification predominantly proceeds through endochondral mechanisms, certain regions—such as those affected by pterygoid processes and specific portions of the greater wing—undergo intramembranous ossification. The formation of the sphenoid bone involves twelve primary ossification centers, which are distributed across the body, including the lesser wings, greater wings, and areas affected by pterygoid processes [1,2].

By the 8th week of fetal development, the ossification center of the greater wing of the sphenoid bone becomes evident, situated between the foramen ovale and the foramen rotundum. The ossification of the lesser wing—referred to developmentally as the orbitosphenoid—commences by the 9th week, originating along the lateral margin of the optic canal. During embryogenesis, the lesser wings derive from prechordal cartilages and orbital prominences, contributing structurally to the anterior cranial base. These elements are critical in the formation of the orbital walls and facilitate the transmission of neural structures into the orbit. The growth of the lesser wing, which encircles the optic nerve, is instrumental in defining the ultimate configuration of the optic canal, while the space demarcated between the greater and lesser wings evolves into the primordial superior orbital fissure [1,2].

According to the study conducted by Zhang et al. [12], the lesser wing develops from two distinct ossification centers that form two arms, surrounding the optic nerve canal. The authors identified these centers as medial and lateral, with ossification occurring earlier in the lateral part than in the medial one. Based on their observations, the fusion of the two ossification centers occurs at the CRL170 stage, corresponding to 20 weeks and 6 days of fetal life. However, in our study, we observed that the ossification centers were already fused by the 17th week of fetal life.

The body of the sphenoid bone ossifies from two pairs of ossification centers, arranged in an anteroposterior direction. The posterior pair is responsible for forming the basisphenoid portion, which develops during the third month of fetal life in the region of the sella turcica. The anterior pair, which appears slightly later, gives rise to the presphenoid portion. An additional laterally located ossification center, responsible for the formation of the sphenoidal lingula and the adjacent segment of the carotid sulcus, rapidly fuses with the basisphenoid part. The ossification centers of the basisphenoid unite during the fourth month of fetal development. At the same time, the bilateral fusion of the ossification center of the lesser wing with the presphenoid portion occurs; however, neither presphenoid parts fuse with the basisphenoid until the eighth month of fetal life. The cartilage separating the anterior and posterior ossification centers of the sphenoid body undergoes resorption before birth or shortly thereafter. A separate ossification center for the lateral part of the pterygoid process appears as early as the second month of fetal life and develops via intramembranous ossification. A similar intramembranous ossification process occurs in a small center located at the apex of the greater wing, whereas the remaining structures of the sphenoid body develop through endochondral ossification [13].

Understanding this process is essential for specialists in anatomy and medicine, as abnormalities in the development of the sphenoid bone may result in cranial malformations and neurological disorders. The improper development of the orbitosphenoid can lead to cranial deformities such as plagiocephaly or orbital anomalies. Plagiocephaly, defined as the asymmetrical deformation of the skull, may occur as either a congenital or acquired condition. It is characterized by the flattening of one side of the head, affecting the shape of the face and the arrangement of cranial bones [14].

Depending on its etiology, plagiocephaly is classified into two main types. Positional (deformational) plagiocephaly is the most commonly observed form, resulting from prolonged external pressure on one side of the head, such as in infants who sleep predominantly in one position. In contrast, synostotic plagiocephaly, which is less frequent, results from the premature fusion of one of the cranial sutures (craniosynostosis), leading to asymmetric cranial growth [14].

Utsunomiya et al. [11] observed significant differences in the morphogenesis of the anterior and posterior parts of the sphenoid bone. Shape changes in the anterior portion persisted for a longer period compared to the posterior part, suggesting greater plasticity in the anterior region, making it more susceptible to remodeling processes. The authors emphasize that this increased plasticity may be associated with a higher risk of craniofacial anomalies involving the anterior part of the sphenoid bone.

Lieberman [15] postulates that a reduction in the length of the sphenoid bone along the midsagittal axis in humans alters the spatial configuration between the cranial base, facial skeleton, and cranial vault, thereby contributing to variations in facial convexity. In contrast, a geometric analysis by Bastir and Rosas [16] suggests that it is not the absolute length of the sphenoid bone, but rather the lateral positioning of its wings relative to the midline of the cranial base, that significantly influences the projection of the maxillary and zygomatic bones.

Moreover, malformations within the sphenoid region are frequently observed in association with various congenital craniofacial anomalies, such as craniosynostosis, cleft lip and palate [17], and Down’s syndrome [18]. This highlights the critical role of the sphenoid bone in the pathogenesis of these conditions.

In a cross-sectional study using 2D ultrasound, involving 386 normal fetuses between 14 and 40 weeks of gestation, Degani et al. [19] measured the maximal transverse width of the sphenoid bone. Additionally, significant positive linear correlations were observed between the length of the sphenoid wing and gestational age, femur length, and biparietal diameter [19].

Levaillant and Mabille [20] compared imaging techniques of the sphenoid bone using computed tomography (CT) and three-dimensional ultrasonography (3D US). The authors noted that acquiring 3D images of the sphenoid bone via ultrasound was less invasive than using CT, while the image quality obtained with both methods was very similar.

Bibliometric data analysis shows that, in recent years, the growth and development of ossification centers of bones forming the cranial base—such as the sphenoid body, frontal bone, and occipital bone—have been extensively evaluated using mathematical modeling approaches [6,7,8,9,10]. However, no researchers to date have conducted linear, planar, or volumetric measurements of the lesser wings of the sphenoid bone. Zhang et al. [12], using a bone-staining method in human fetuses, measured the distance between the ossification centers of the right and left lesser wings, defined as the distance between their most peripheral borders. In fetuses with a crown–rump length (CRL) of 120 mm, this distance measured 6.37 ± 0.34 mm, whereas in those with a CRL of 170 mm, it measured 6.91 ± 1.13 mm.

Accordingly, the present study constitutes the first detailed characterization of the linear, planar, and volumetric parameters of the ossification centers of the lesser wing of the sphenoid bone, accompanied by a modeled analysis of their growth dynamics in human fetuses between 18 and 30 weeks of gestation.

All analyzed parameters of the ossification centers of the lesser wings increased proportionally with fetal age in weeks, indicating commensurate growth between 18 and 30 weeks of gestation. The appropriate selection of the best-fit function was based on the highest value of the coefficient of determination (R^2^), which effectively illustrates both the growth of the studied parameter and the degree of fit to the function. In our study, the highest coefficients of determination ranged from 0.95 to 0.98, indicating an excellent fit of the growth model to all analyzed parameters of the ossification centers of the lesser wings.

The absence of significant sex-related differences in the development of the sphenoid bone in fetuses is consistent with findings reported in the existing literature. Morimoto et al. [21], using computed tomography to examine formalin-fixed fetuses, did not demonstrate the presence of sexual dimorphism in the fetal cranium. Similar conclusions can be drawn from our previous studies, in which no sex differences were found in the development of the sphenoid bone body [6].

To date, the scientific literature lacks comprehensive data on the quantitative anatomy of the fetal skeleton at defined gestational ages based on computed tomography imaging. In particular, existing medical reports do not provide information regarding the dimensions of the ossification centers of the lesser wings of the sphenoid bone in human fetuses, thereby limiting the possibility of conducting an in-depth analysis of this aspect of cranial development. Consequently, the findings of the present study may offer a valuable contribution to the understanding of fetal skeletal growth by introducing novel quantitative data with potential diagnostic and research applications. A detailed understanding of the growth patterns of individual cranial bones in the human fetus may prove useful across multiple disciplines, including anatomy, anthropology, forensic medicine, orthodontics, radiology, obstetrics, pediatrics, orthopedics, and reconstructive surgery [6,7,8,9,10].

### Limitations of the Study

The quantitative data obtained from the ossification centers of the lesser wings of the sphenoid bone may serve as a valuable reference for monitoring normal fetal development and for use in the screening and early diagnosis of congenital anomalies. Nonetheless, it is important to acknowledge the principal limitations of the present study, namely the relatively narrow gestational age range of the analyzed specimens (18 to 30 weeks) and the modest sample size (*n* = 37).

## 5. Conclusions

1. No sex-related differences were observed in any of the morphometric parameters of the ossification centers of the lesser wings of the sphenoid bone.
2. The developmental dynamics of all studied parameters of the ossification centers of the lesser wings of the sphenoid bone increased proportionally with gestational age in weeks.
3. The obtained morphometric data on the ossification centers of the lesser wings of the sphenoid bone may serve as age-specific reference values, supporting gestational age estimation and aiding in the early ultrasonographic diagnosis of craniofacial developmental anomalies. Further research is recommended to expand our understanding of their growth patterns and potential clinical relevance.

## Figures and Tables

**Table 1:** Age, number, and sex of the fetuses studied.

Month	GA (Weeks)				Crown–Rump Length (mm)	Number of Fetuses	
		Mean	SD	Max.	N	♂	♀
V	18	133.33	5.77	140.00	3	1	2
	19	146.50	2.89	150.00	4	2	2
	20	161.00	2.71	165.00	4	2	2
VI	21	173.67	2.31	175.00	3	2	1
	22	184.67	1.53	186.00	3	1	2
	23	198.67	2.89	202.00	3	1	2
	24	208.00	3.56	213.00	4	1	3
VII	25	214.00		214.00	1	0	1
	26	229.00	5.66	233.00	2	1	1
	27	240.33	1.15	241.00	3	3	0
	28	249.50	0.71	250.00	2	0	2
VIII	29	253.00	0.00	253.00	2	0	2
	30	262.67	0.58	263.00	3	2	1
					Total	37	16

**Table 2:** Intra-class correlation coefficient (ICC) values for inter-observer recurrence.

Parameter	ICC
Right length of the anterior (orbital) part	0.995 *
Right length of the posterior (sphenoid) part	0.996 *
Right width	0.996 *
Right projection surface area	0.999 *
Right volume	0.998 *
Left length of the anterior (orbital) part	0.996 *
Left length of the posterior (sphenoid) part	0.996 *
Left width	0.997 *
Left projection surface area	0.999 *
Left volume	0.998 *

Intra-class correlation coefficients marked with * are statistically significant at *p* < 0.0001.

**Table 3:** Length of anterior and posterior parts, width, projected surface area, and volume of right ossification centers of lesser wing of sphenoid bone.

Month	GA (Weeks)	N	Statistically Significant Effect of Sex	Right Ossification Center of the Lesser Wings of Sphenoid Bone									
				Length of the Anterior (Orbital) Part (mm)		Length of the Posterior (Sphenoid) Part (mm)		Width (mm)		Projection Surface Area (mm^2^)		Volume (mm^3^)	
				Mean	SD	Mean	SD	Mean	SD	Mean	SD	Mean	SD
V	18	3	*p* > 0.05	3.19	0.03	3.29	0.05	3.60	0.10	10.81	0.47	11.79	0.62
	19	4		3.31	0.06	3.62	0.10	3.91	0.12	12.75	0.50	14.45	0.70
	20	4		3.51	0.07	3.96	0.17	4.31	0.11	14.22	0.71	16.82	0.91
VI	21	3	*p* > 0.05	3.67	0.04	4.41	0.11	4.67	0.13	16.96	0.92	20.24	1.18
	22	3		3.85	0.11	4.76	0.14	5.04	0.14	19.53	0.98	23.57	1.28
	23	3		4.26	0.15	5.04	0.14	5.31	0.10	21.93	0.95	26.68	1.26
	24	4		4.69	0.11	5.30	0.10	5.72	0.14	24.98	1.07	30.92	1.54
VII	25	1	*p* > 0.05	4.90		5.47		5.94		26.72		33.40	
	26	2		5.03	0.08	5.57	0.04	6.09	0.08	27.83	0.50	35.48	0.83
	27	3		5.35	0.13	5.68	0.06	6.37	0.11	29.88	0.84	39.25	1.40
	28	2		5.63	0.05	5.83	0.08	6.63	0.08	31.70	0.64	43.28	1.55
VIII	29	2	*p* > 0.05	5.81	0.10	5.96	0.06	6.83	0.05	33.59	0.56	48.21	1.52
	30	3		6.05	0.10	6.19	0.11	6.96	0.04	35.53	0.84	52.70	1.61

**Table 4:** Length of anterior and posterior parts, width, projected surface area, and volume of left ossification centers of lesser wing of sphenoid bone.

Month	GA (Weeks)	N	Statistically Significant Effect of Sex	Left Ossification Center of the Lesser Wings of Sphenoid Bone									
				Length of the Anterior (Orbital) Part (mm)		Length of the Posterior (Sphenoid) Part (mm)		Width (mm)		Projection Surface Area (mm^2^)		Volume (mm^3^)	
				Mean	SD	Mean	SD	Mean	SD	Mean	SD	Mean	SD
V	18	3	*p* > 0.05	3.22	0.04	3.26	0.05	3.87	0.10	11.32	0.39	12.12	0.70
	19	4		3.40	0.05	3.59	0.09	4.22	0.17	12.63	0.47	14.18	0.68
	20	4		3.56	0.05	3.92	0.16	4.63	0.10	13.71	0.33	16.11	0.45
VI	21	3	*p* > 0.05	3.70	0.10	4.37	0.12	4.93	0.10	15.01	0.43	17.71	0.51
	22	3		3.90	0.10	4.66	0.11	5.24	0.10	16.74	0.80	20.15	1.04
	23	3		4.30	0.16	4.97	0.13	5.54	0.09	19.60	1.01	23.78	1.33
	24	4		4.71	0.11	5.26	0.10	5.89	0.13	22.89	1.03	28.34	1.56
VII	25	1	*p* > 0.05	4.92		5.42		6.14		24.97		31.46	
	26	2		5.08	0.09	5.51	0.03	6.26	0.06	26.09	0.67	33.27	1.04
	27	3		5.38	0.15	5.65	0.07	6.47	0.07	28.70	0.98	38.38	1.75
	28	2		5.68	0.03	5.77	0.05	6.62	0.03	30.91	0.40	43.43	1.22
VIII	29	2	*p* > 0.05	5.85	0.08	5.93	0.06	6.74	0.04	32.48	0.72	47.10	1.05
	30	3		6.11	0.10	6.15	0.11	6.87	0.05	34.48	0.82	50.92	1.59

**Table 5:** Anterior and posterior inter-center diameters of the ossification centers of the lesser wings of the sphenoid bone.

Month	GA (Weeks)	N	Statistically Significant Effect of Sex	N	Anterior (Orbital) Inter-Center Diameter (mm)		Posterior (Sphenoid) Inter-Center Diameter (mm)	
					Mean	SD	Mean	SD
V	18	3	*p* > 0.05	3	6.21	0.04	7.66	0.07
	19	4		4	6.32	0.04	7.76	0.04
	20	4		4	6.42	0.04	7.85	0.04
VI	21	3	*p* > 0.05	3	6.54	0.03	7.95	0.03
	22	3		3	6.62	0.04	8.01	0.05
	23	3		3	6.71	0.04	8.13	0.05
	24	4		4	6.80	0.04	8.30	0.05
VII	25	1	*p* > 0.05	1	6.87		8.40	
	26	2		2	6.92	0.03	8.48	0.01
	27	3		3	7.01	0.04	8.53	0.04
	28	2		2	7.09	0.03	8.60	0.01
VIII	29	2	*p* > 0.05	2	7.17	0.02	8.62	0.01
	30	3		3	7.23	0.03	8.65	0.01

## Data Availability

The original contributions presented in this study are included in the article. Further inquiries can be directed to the corresponding author.
